# Structural Characterization, Technofunctional and Rheological Properties of Sesame Proteins Treated by High-Intensity Ultrasound

**DOI:** 10.3390/foods12091791

**Published:** 2023-04-26

**Authors:** Osman Gul, Furkan Turker Saricaoglu, Ilyas Atalar, Latife Betul Gul, Fatih Tornuk, Senay Simsek

**Affiliations:** 1 Department of Food Engineering, Faculty of Engineering and Architecture, Kastamonu University, 37150 Kastamonu, Turkey; osmangul@kastamonu.edu.tr; 2 Department of Food Engineering, Faculty of Engineering and Natural Sciences, Bursa Technical University, 16310 Bursa, Turkey; 3 Department of Food Engineering, Faculty of Agriculture, Eskisehir Osmangazi University, 26160 Eskisehir, Turkey; 4 Department of Food Engineering, Faculty of Engineering, Giresun University, 28200 Giresun, Turkey; 5 Department of Food Engineering, Faculty of Chemical and Metallurgical Engineering, Yildiz Technical University, 34349 Istanbul, Turkey; 6 Department of Food Science & Whistler Center for Carbohydrate Research, Purdue University, West Lafayette, IN 47907, USA

**Keywords:** sesame protein, high-intensity ultrasound, technofunctional properties, structural properties, rheological properties

## Abstract

Plant-derived proteins, such as those from sesame seeds, have the potential to be used as versatile food ingredients. End-use functionality can be further improved by high-intensity ultrasound treatments. The effects of high-intensity ultrasound on the properties of sesame protein isolates from cold-pressed sesame cake were evaluated. The SDS-PAGE demonstrated no significant changes in the molecular weight of proteins. Ultrasound treatments resulted in decreased particle size with a more uniform distribution, resulting in the exposure of hydrophobicity and free −SH groups and increased zeta potential. Although FTIR spectra of proteins were similar after ultrasonication, a partial increase in the intensity of the amide A band was observed. The ultrasound significantly (*p* < 0.05) affected the secondary structure of proteins. While optical micrographics revealed a dispersed structure with smaller particles after treatments, microstructural observations indicated more rough and irregular surfaces. Water solubility was improved to 80.73% in the sample subjected to 6 min of ultrasonication. Sesame protein solutions treated for 4 and 6 min exhibited viscoelastic structure (storage modulus (G′) > loss modulus (G′′)). In addition, the gelation temperature of proteins decreased to about 60–65 °C with increasing treatment time. Overall, ultrasound is a useful technique for the modification of sesame protein isolates.

## 1. Introduction

In recent years, interest in plant-derived proteins has increased due to health benefits like reducing the risk of developing metabolic syndrome, preventing the incidence of cancers, and regulating weight and diabetes, as well as higher availability, lower cost, and consumer preference, such as religious or other beliefs [1,2]. Additionally, studies have shown that the potential for plant-derived proteins to be used as versatile food ingredients that have good functional characteristics like emulsification, gelation, and foaming is quite high [3,4]. Sesame (*Sesamum indicum* L.) is an important crop for edible oil, food, and animal feed industries, and is mainly cultivated in tropical and subtropical agro-ecologies, with global production of about 7.25 million tons in 2020 [5]. Sesame consumption is also steadily increasing due to its nutritional characteristics, such as its healthy oil, protein, vitamins, minerals, and fiber [6]. Sesame seed is mainly used for oil production and sesame cake is obtained as the primary by-product of the sesame oil extraction process, which is generally used as feed due to its high protein content [7]. Sesame cake is also directly discarded as industrial waste, thus causing waste of protein resources. Generally, by-products of the oil industry are gaining importance due to their relative abundance and low cost [1]. Sesame cake contains 35–40% protein rich in essential amino acids accounting for 30% of total amino acids [8]. Moreover, sesame protein, an ideal plant protein resource, is rich in sulfur-containing amino acids like methionine and tryptophan, which are easily digested and absorbed by the human body [9]. Although it has high nutritional value, it is necessary to improve its functional properties, such as water/oil holding, gelation, emulsification, viscosity, etc., for wide utilization in the food industry [8].

Technofunctional properties of plant-derived proteins, which are the key factors for manufacturers, can be affected by various factors, such as particle size, molecular structure, ionic forces, pH, extraction methods, and other food components [1]. Therefore, recent studies have been focused on modifying proteins to improve their functional properties by changing these factors using several techniques, including ultrasound, high-pressure homogenization, extrusion cooking, cold plasma, pH-shifting, and heat treatment [10]. Ultrasound, which is an emerging physical processing technology, is used to change/modify the structure and functional characteristics of proteins. Ultrasound reduces the particle size of aggregates and changes molecular structure through acoustic cavitation, including water jets, shock waves, and high shear by micro- and macro-streaming during the ultrasonication process [11,12]. Ultrasound can be categorized as low- or high-intensity. High-intensity sonication technology (ultrasonication) has an intensity greater than 1 W/cm^2^ at a frequency between 20 and 500 kHz [13], which is mostly used for the modification of proteins such as amaranth, pumpkin seed, potato, etc. [4,14,15]. However, few articles have been published on the functional properties of sesame protein after a high-intensity sonication process. Yang, Xu, Fu, Cai, Xia, Guan, Zou, and Sun [2] studied the effects of ultrasonic-assisted extraction on the physicochemical properties of sesame proteins. Similarly, a study conducted by Görgüç et al. [16] focused on the extraction and characterization of sesame protein via the simultaneous effects of vacuum and ultrasound. As a technological approach, improving and/or enhancing the technofunctional properties of plant-based proteins is of great importance for the food industry. This importance is noteworthy in terms of their potential as substitutes for animal-derived proteins. In this context, the fact that ultrasound applications can be applied at industrial scales reveals the importance and practical potential of this study. Yet knowledge of the effects of ultrasonication, which is directly applied to the sesame protein solution, on the structural, technofunctional, and rheological properties of sesame protein has not been investigated. Therefore, the hypothesis of this study is that high-intensity ultrasound will have an effect on the structural changes and resultant technofunctional and rheological properties of sesame protein isolate extracted from cold-pressed sesame cake.

## 2. Materials and Methods

### 2.1. Materials

White sesame samples were supplied by Aslan Sesame and Tahin Food Ltd. Co. (Eskisehir, Turkey) and the sesame oil was removed from sesame by cold-press oil extraction using a cold-press machine (Kocmaksan, Izmir, Turkey). The cold-pressed sesame cake was ground with a Waring blender (Waring-80011 S, Stamford, CT, USA) and sieved at 0.25 mm. The sesame cake powder obtained after pressing contained 4.3% moisture, 38.7% protein, 11.56% lipid, 6.63% ash, and 38.81% carbohydrate. The cake powder was used for sesame protein extraction. All chemicals and solvents used were of analytical grade and purchased from Merck (Darmstadt, Germany) and Sigma-Aldrich (Saint Louis, MO, USA).

### 2.2. Preparation of Sesame Protein Isolate

Protein extraction from sesame cake powder was carried out using the method proposed by Gandhi and Srivastava [17], with partial modification. Firstly, sesame powder was mixed with distilled water at a ratio of 1:9 (*w/v*), and the mixture was homogenized at 9000 rpm for 3 min with Ultra-Turrax (Daihan, HG-15D, Gangwon-do, Republic of Korea). The pH of the suspension was adjusted and maintained at 10.0 with 1 mol/L NaOH, and the suspension was stirred at 400 rpm for 1 h at room temperature. In order to separate the soluble proteins from the insoluble parts, the slurry was centrifuged (Nuve, NF-800R model, Ankara, Turkey) at 9000 rpm at 4 °C for 10 min. The supernatant was collected in a beaker, and the pH was adjusted to 4.5 with 1 mol/L HCl, allowing the proteins to precipitate at an isoelectric pH. Then it was centrifuged again under the same conditions, and the precipitate was collected. The collected precipitate was frozen at −24 °C for 24 h, and then lyophilized using a freeze dryer (Teknosem, Toros TRS-4/4, Istanbul, Turkey) at −56 °C at 10^−3^ mbar pressure. Dried sesame proteins were ground into a fine powder and stored in glass jars at 4 °C until analysis. The protein content of the sesame protein isolate was found to be 90.33%, which was determined using the Kjeldahl method [17].

### 2.3. High-Intensity Ultrasound Treatment

Sesame protein isolate was mixed with distilled water to contain 4% protein by weight, and the mixture’s pH was adjusted to 9.0 with 1 mol/L NaOH to achieve a high solubility. After continuously stirring for 2 h at room temperature, ultrasonic treatments were performed using an ultrasonic processor (JP Selecta, CY-500, Barcelona, Spain) with a 0.67 cm diameter titanium probe at an ultrasonic power of 95% amplitude, and a constant frequency of 20 kHz for 2, 4, and 6 min. A sample without sonication treatment served as a control. To avoid an excessive increase in temperature during and after the sonication process, the protein solution was placed in a beaker that was placed in an ice–water mixture. The ultrasonic treated protein samples were frozen at −24 °C for 24 h and then freeze-dried in a lab type freeze dryer (Teknosem, Toros TRS-4/4, Istanbul, Turkey) and kept at 4 °C for further analysis.

### 2.4. Physicochemical Properties

#### 2.4.1. Sodium Dodecyl Sulfate-Polyacrylamide Gel Electrophoresis (SDS-PAGE)

SDS-PAGE analyses of sesame proteins to determine the molecular weight of protein fractions were performed using the method proposed by Laemmli [18], using 12% and 5% acrylamide slab gels for separating and stacking, respectively. A total of 200 µL of sesame protein solution (25 mg/mL) was mixed with 200 µL of buffer solution containing 0.125 mol/L Tris-HCl (pH 6.8), 4% SDS, 20% glycerol, and 0.5% mercaptoethanol, and the solution was heated in a boiling water bath for 5 min. After the cooling, 15 µL of each sample was injected into stacking gel wells mounted in electrophoresis equipment (Mini-PROTEAN II, Bio-Rad, Hercules, CA, USA), after bromphenol blue (10 µL) was added. The SDS-PAGE was run at 100 V for 90 min to complete the separation of subunits and/or peptides. The gels were stained overnight using a solution consisting of 0.1% coomassie brilliant blue, 40% methanol, and 7% glacial acetic acid and then de-stained by a methanol and acetic acid mixture. A protein marker with a 10–250 kDa molecular weight was applied as the standard (Bio-Rad).

#### 2.4.2. Particle Size and Zeta Potential

Particle size distributions and zeta potential of sesame proteins before and after sonication treatment were carried out with a laser diffraction particle size analyzer (Malvern Instruments, Mastersizer 3000, Worcestershire, UK) and a zetasizer Nano ZS90 (Malvern Instruments, Worcestershire, UK), respectively. The protein suspensions were prepared with ultrapure water to a concentration of 0.01 mg/mL using a magnetic stirrer for 2 h, and the measurement was conducted in triplicate.

#### 2.4.3. Fourier Transform Infrared Spectroscopy (FTIR)

Possible change in the secondary structures of sesame protein modified by ultrasonication technique was carried out by FTIR (Perkin Elmer, Spectrum Two model, Boston, MA, USA) analysis. For this purpose, powder samples were analyzed in the wavelength range of 4000–650 cm^−1^ with a resolution of 4 cm^−1^. In addition, peaks (amide-I) of samples in the wavelength range of 1700–1600 cm^−1^ were subjected to a self-deconvolution process to determine the effect of the modification process on the secondary structural properties (α-helix, β-sheet, β-turn, and random coil) of proteins.

#### 2.4.4. Optical and Scanning Electron Microscopy (SEM)

To observe microstructure of protein suspensions, microstructure images were captured using a Leica DFC295 Optical Microscope (Leica Microsystems, Heerbrugg, Switzerland). Protein solutions (50 µL) were transferred onto a microscope glass slide, covered with another slide, and observation was carried out at 40× magnification.

The effects of the modification process on the microstructural properties of sesame proteins were determined with an SEM (FEI, Quanta FEG 250, Hillsboro, OR, USA). After the protein samples were coated with gold-palladium, the visualization process was carried out with a magnification of 5000× at 5 kV acceleration voltages.

#### 2.4.5. Protein Solubility

The water solubility determination of control and modified sesame proteins was carried out according to the method suggested by Klompong et al. [19]. Sesame protein (200 mg) was diluted with 20 mL of distilled water, and the solution was stirred for 1 h at room temperature. The protein suspensions were then centrifuged at 7500 rpm for 15 min. The protein content of the supernatant was determined using the Biuret method [20]. An amount of 1 mL of supernatant was mixed with 1 mL of Biuret reagent, and the absorbance of the mixture was read at 550 nm against the Biuret reagent as a blank using a spectrophotometer (Shimadzu, 1800, Kyoto, Japan). The solubility was calculated with the following formula using bovine serum albumin as a standard.

(1) $$\mathrm{Solubility}\left(\%\right)=\frac{\mathrm{Protein}\;\mathrm{content}\;\mathrm{of}\;\mathrm{supernatant}}{\mathrm{Total}\;\mathrm{protein}\;\mathrm{content}\;\mathrm{of}\;\mathrm{the}\;\mathrm{sample}}\times 100$$

#### 2.4.6. Surface Hydrophobicity

The surface hydrophobicity of protein suspensions was determined by 1-anilino-8-naphathalene-sulfonate (ANS) fluorescent according to Arzeni et al. [21]. Sesame protein samples were diluted to a concentration in the range of 0.025–0.2 mg/mL with 0.1 mol/L phosphate buffer (pH 7.0). Then 2.5 mL of protein suspension was added to 12.5 µL of 1-anilino-8-naphathalene-sulfonate (ANS) (dissolved in phosphate buffer) and held for a 20 min reaction at room temperature in the dark. The fluorescence intensities of the mixture were measured at a 390 nm excitation wavelength, 470 nm emission wavelength, and 5 nm slit width at 25 °C with a fluorescent spectrophotometer (FluoroMax-4, Horiba Scientific, Kyoto, Japan). The initial slope of the relative fluorescence intensity (RFI) vs. the protein content of the dilutions was used to compute the surface hydrophobicity index. RFI was calculated as: RFI = (F − F_0_)/F_0_, where F and F_0_ are the fluorescence reading of the protein–ANS conjugate and the protein-free ANS solution, respectively [22].

#### 2.4.7. Free and Total Surface Sulfhydryl (−SH) Groups

The effects of ultrasonication treatments on −SH groups of sesame proteins were determined by modifying the method of Ellman [23]. To determine free −SH groups, buffer (A) solution was prepared with 0.086 mol/L Tris, 0.09 mol/L glycine, and 4 mmol/L ethylenediamine-tetra acetic acid disodium salt at pH 7.0. Control and modified protein powders were mixed with buffer (A) solution to contain 0.2% protein and centrifuged at 4 °C for 15 min at 8000× *g*. After centrifugation, 3 mL of the supernatant was mixed with 0.03 mL of 4 mg/mL of Ellman’s agent (5’,5-dithiobis 2-nitrobenzoic acid, DTNB) dissolved in buffer (A) and then incubated at 25 °C for 15 min. Free −SH groups were determined by reading absorbance at a 412 nm wavelength using a spectrophotometer [24]. In order to determine the total −SH groups, 6 mol/L urea and 0.5% (*w/v*) SDS were added to the buffer (A) solution and the above-mentioned procedures were applied exactly [21]. Free and total −SH groups were calculated using the following equation:

(2) $$-SH\left(µ\frac{mol}{g}\right)=(73.53\times A_{412}\times D)/C$$

where *A*_412_ refers to the absorbance at 412 nm; *C* refers to the concentration of protein solution (mg/mL); and *D* refers to the dilution factor. To calculate micromoles of SH/g of soluble solids, a molar extinction coefficient of 1.36 × 10^4^ was used.

### 2.5. Technofunctional Properties

#### 2.5.1. Emulsifying Properties

The emulsion activity index (EAI) and stability index (ESI) of the proteins were measured as described by Pearce and Kinsella [25]. The protein sample (300 mg) was mixed with 30 mL of distilled water, and 10 mL of sunflower oil was added to the mixture. Then the mixture was homogenized at 20,000 rpm for 1 min. Immediately after emulsion formation, and 10 min later, 50 µL of the emulsion sample was mixed with 5 mL of 0.1% SDS solution, and the absorbance was read at 500 nm wavelength. EAI and ESI were determined as follows:

(3) $$EAI=\frac{2\times 2.303\times A_{0}}{0.25\times \mathrm{protein}\;\mathrm{weight}\;(g)}$$

(4) $$ESI=\frac{A_{10}\times ∆t}{∆A}$$

where *A*_0_ and *A*_10_ refer to the absorbance at 0 min and 10 min respectively; Δ*t* = 10 min, and Δ*A* refer to the difference between the initial absorbance and absorbance after 10 min.

#### 2.5.2. Foaming Properties

To determine the foaming capacity (FC) and stability (FS) of proteins, 300 mg of protein sample was mixed with 30 mL of distilled water and the mixture was transferred to a 100 mL measuring cylinder. The mixture was homogenized with an Ultra-Turrax at 14,000 rpm for 3 min, and the volume was measured. After 30 min of storage, the volume was measured again. The foaming capacity and stability were calculated using Equations (5) and (6), respectively [26].

(5) $$Foaming\;capacity\;\left(\%\right)=\frac{V_{2}-V_{1}}{V_{1}}$$

(6) $$Foaming\;stability\;\left(\%\right)=\frac{V_{3}-V_{1}}{V_{2}}$$

where *V*_1_, *V*_2_, and *V*_3_ refer to the volume of protein dispersion before and after homogenization, and after 30 min of storage, respectively.

#### 2.5.3. Water- and Oil-Holding Capacity

Water- and oil-holding capacities of sesame proteins were analyzed following the method reported by Ogunwolu, Henshaw, Mock, Santros, and Awonorin [26]. For this purpose, 100 mg of protein sample and 2 mL of distilled water or 1 mL of sunflower oil were placed in a pre-weighted centrifuge tube and vortexed. The dispersion obtained was then centrifuged at 1800 rpm for 20 min at 25 °C. After removing the supernatant, the tubes were weighed and water- and oil-holding capacity were calculated as grams of water or oil absorbed per gram of protein, respectively.

### 2.6. Rheological Properties

Rheological experiments of sesame protein suspensions were performed by using a rheometer (Anton Paar, MCR 302 model, Graz, Austria) equipped with a conical-plate geometry with a conical angle of 2° and a diameter of 25 mm; the gap size was 0.106 mm. A Peltier system was used to control the temperature of the rheometer. Protein suspensions (4 g/100 mL) were kept for 2 min at 25 °C to equilibrate and all measurements were conducted under a temperature of 25 °C in duplicate. The data of all rheological measurements were analyzed with the software Rheoplus/32 v2.81 (Anton Paar).

#### 2.6.1. Steady Shear Tests

The flow behavior of sesame protein suspensions treated with ultrasound was measured using shear stress against shear rates over a range of 1–100/s. Data were fitted to the Ostwald–de Waele model for describing the relationship between shear stress and shear rate:

(7) $$\tau =K\times {\dot{\gamma }}^{n}$$

where *τ* refers to the shear stress (Pa), *K* refers to the consistency index (Pas*^n^*), $\dot{\gamma }$ refers to the shear rate (s^−1^), and *n* refers to the flow behavior index (dimensionless).

#### 2.6.2. Dynamic Shear Tests

Dynamic shear tests were carried out to determine the viscoelastic properties of sesame proteins. For this purpose, a stress sweep test was carried out at a constant frequency of 0.681 rad/s in the range of 0.01–10 Pa to determine the linear viscoelastic region (LVR) of the solution. Then frequency sweep tests were performed at a frequency in the range of 0.681–46.42 rad/s at 0.2 Pa, which is in the LVR (data not shown). The power law model was used to analyze storage (*G*′) and loss (*G*′′) modulus changes depending on the frequency range:

(8) $$G^{\prime }=K^{\prime }{\left(\omega \right)}^{n^{\prime }}$$

(9) $$G^{\prime \prime }=K^{\prime \prime }{\left(\omega \right)}^{n^{\prime \prime }}$$

where *K*′ and *K*″ refer to power law modulus constants (Pas*^n^*), *n*′ and *n*″ may be referred to as the frequency exponents, and *ω* refers to the angular frequency (rad/s).

#### 2.6.3. Temperature Sweep Tests

The effects of ultrasound on the thermal properties of sesame proteins were determined by a temperature sweep test at constant pressure (0.2 Pa) and frequency (0.681 rad/s) within the LVR over a temperature range of 10–95 °C with a heating rate of 10 °C/min. Protein suspension samples were loaded to the bottom plate, and silicone oil was used to prevent evaporation from the sample during heating. *G*′ and *G*′′ modules of proteins were recorded depending on the temperature increase, and gelation temperatures were determined.

### 2.7. Statistical Analysis

All experiments were conducted in triplicate, and the result was expressed as mean ± standard deviation using the SPSS statistical package program version 21.0 (SPSS Inc., Chicago, IL, USA). A one-way ANOVA was performed to compare the means, and the differences between the samples were determined using Duncan’s multiple range test based on a significance level of *p* < 0.05.

## 3. Results and Discussion

### 3.1. SDS-PAGE

**Figure 1:**
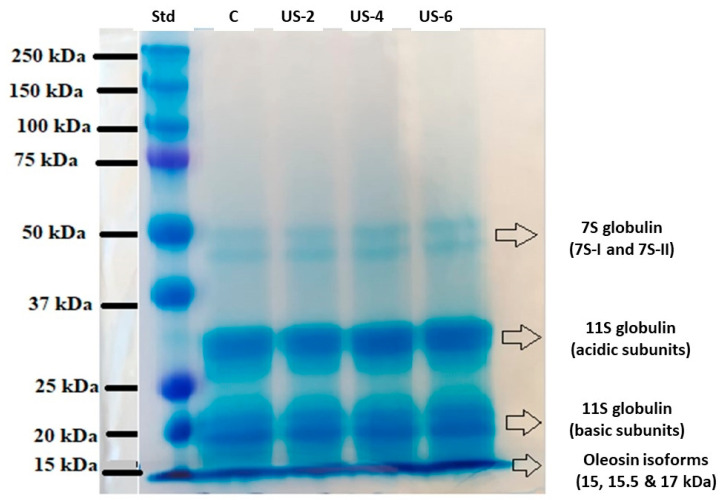
SDS-PAGE profiles of untreated and high-intensity-ultrasound-treated sesame protein isolates. (C, untreated (control); US−2, US−4, and US−6, high-intensity ultrasound treatment for 2, 4, and 6 min, respectively).

The molecular weight profiles of sesame protein fractions were determined using electrophoresis, and SDS-PAGE profiles are shown in Figure 1. Four intense bands with molecular weight ranges of 50–37 kDa (as two subfractions), 37–25 kDa (as two subfractions), 20 kDa (as sub-fraction), and <15 kDa were observed for all sesame protein subunits. According to the literature, 80–90% of the total sesame protein consists of water-soluble 11S globulins linked by disulfide bonds and 2S albumin with a molecular weight of 20–13 kDa [27]. Additionally, it has been reported that 7S globulins constitute 1–2% of total sesame proteins [28]. Achouri, Nail, and Boye [9] observed the protein bands between 10 to 100 kDa in the aqueous sesame extract, and they reported that the extracts obtained with ammonium sulfate solutions at different concentrations contain 7S globulin and 2S albumin bands in 140 kDa.

As was shown in Figure 1, no obvious change was noted in the main bands of ultrasonicated samples compared to the control sample, indicating that the ultrasound process did not change the molecular weights of sesame proteins. This shows that the peptide bonds are not changed due to the sonication process. Similarly, in other studies in the literature, it has been noted that there is no change in the molecular weight profiles of walnut [29] and squid [30] protein isolates after ultrasound treatment. However, Malik, Sharma, and Saini [31] reported that both probe and bath sonication processes caused a reduction in molecular weight of sunflower protein isolates due to high shear stress and turbulence effect of ultrasound, which resulted in the splitting of protein molecular structures. The difference in the molecular weight distributions of the proteins exposed to ultrasound may be due to the applied ultrasound treatment parameters and the differences in the protein sources.

### 3.2. Particle Size and Zeta Potential

**Figure 2:**
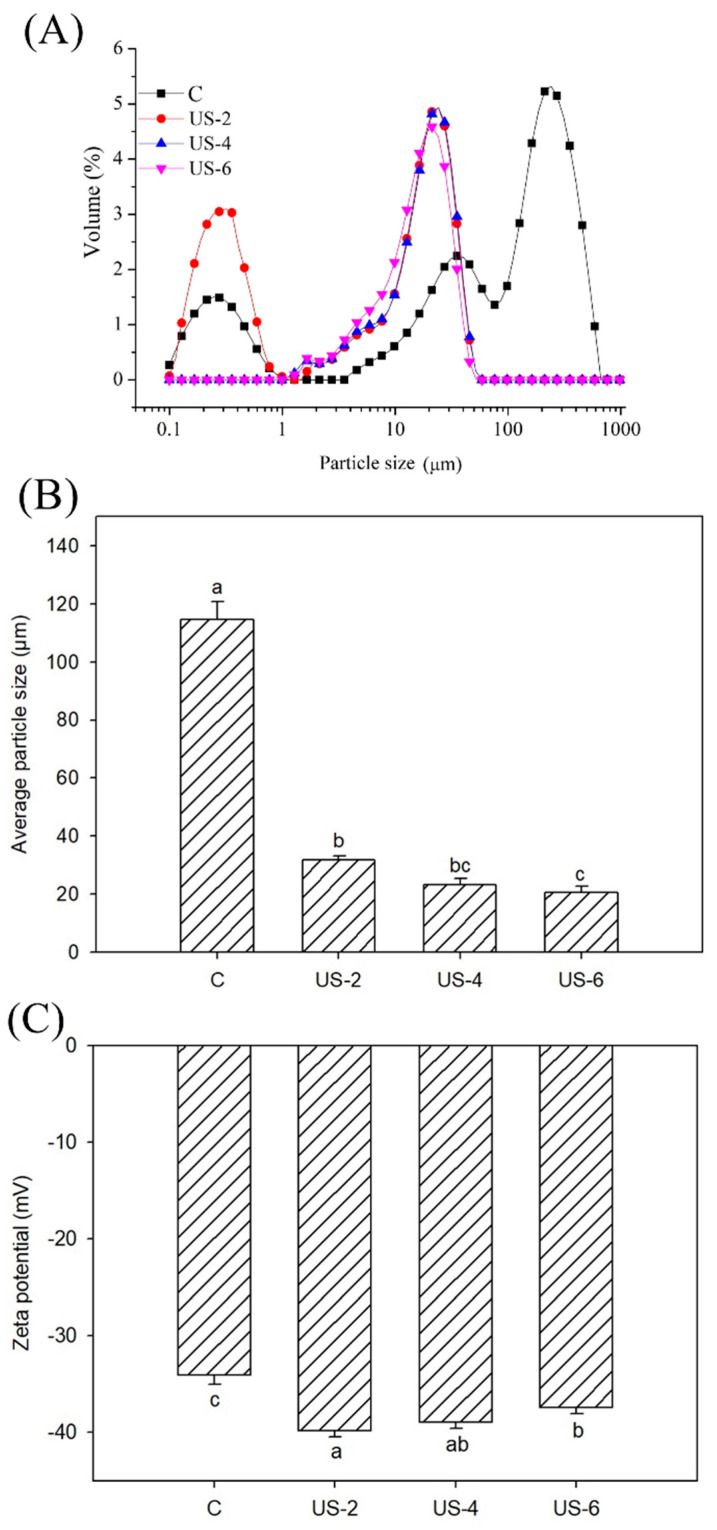
Particle size distribution (**A**), average particle size (**B**), and zeta potential (**C**) of untreated and high-intensity-ultrasound-treated sesame protein isolates. (C, untreated (control); US−2, US−4, and US−6, high-intensity ultrasound treatment for 2, 4, and 6 min, respectively). Columns in the same graph with the same letter are not significantly different.

Particle size distribution profiles of sesame protein suspensions subjected to ultrasound treatment are shown in Figure 2A. The control and samples treated with ultrasound for 2 min exhibited bimodal size distribution in suspension, while the application of ultrasound for 4 and 6 min resulted in more homogeneity with a unimodal distribution. Compared to the control sample, the particle size distribution of ultrasonicated samples became narrower in distribution, which is possibly ascribed to the swelling of protein particles or to the formation of aggregates [32].

Figure 2b shows the results of particle size values of sesame protein isolate treated and untreated by ultrasound. The application of ultrasound reduced the interactions between the particles and caused significant (*p* < 0.05) reductions in particle size compared to the control sample. Ding et al. [33] stated that the reduction of particle size during sonication treatment possibly related to the fact that non-covalent interactions and violent collisions between protein aggregates are damaged by the turbulent effect, cavitation, and strong shearing force from ultrasound intensity. Our results are compatible with earlier experiments indicating that ultrasound led to a reduction in particle size of some plant and animal proteins [31,34]. However, an increase in sonication time did not present considerable changes, probably due to the highly aggregated structure of the insoluble component of protein isolate. The increasing sonication time probably caused an increase in the aggregation of protein particles. Similarly, Yanjun et al. [35] indicated that there was no significant reduction in particle size as the time of sonication was prolonged.

The zeta potential of sesame protein isolates treated with ultrasound was also studied because it is usually used to characterize the stability of the solution system. The magnitude of the absolute value of the zeta potential in the solution system indicates the repulsive force between the molecules. This means that the tendency for aggregation in the system would reduce [36]. Figure 2c shows the change in the zeta potential of sesame protein during ultrasound treatment. The zeta potentials of all samples were negative, indicating the presence of more negatively charged amino acids than positively charged ones on the protein surface [37]. The zeta potential of protein isolates increased with the applied sonication process, indicating that it was beneficial to enhance the stability of the protein solution due to the good electrostatic repulsion. Our results are consistent with studies performed by Nazari, Mohammadifar, Shojaee-Aliabadi, Feizollahi, and Mirmoghtadaie [37] and Zhang et al. [38], in which an increase in the absolute zeta potential of protein solutions after ultrasound treatment was determined. The increase in the zeta potential is due to mechanical forces such as cavitation that occur during sonication. Polar groups in the inner regions of the protein move to the surface of protein molecules via the effect of cavitation. Thus, there is more net charge in the modified protein solution, and electrostatic interactions form more tightly bound complexes, improving the stability and distribution of the complex [39]. The highest value of zeta potential (39.8 mV) was detected in the sample sonicated for 2 min, and an increase in time caused a decrease in the zeta potential of samples. This reduction in zeta potential could be related to the masking of polar regions on the protein surface as a result of the formation of aggregates [40].

### 3.3. Fourier Transform Infrared Spectroscopy (FTIR)

**Figure 3:**
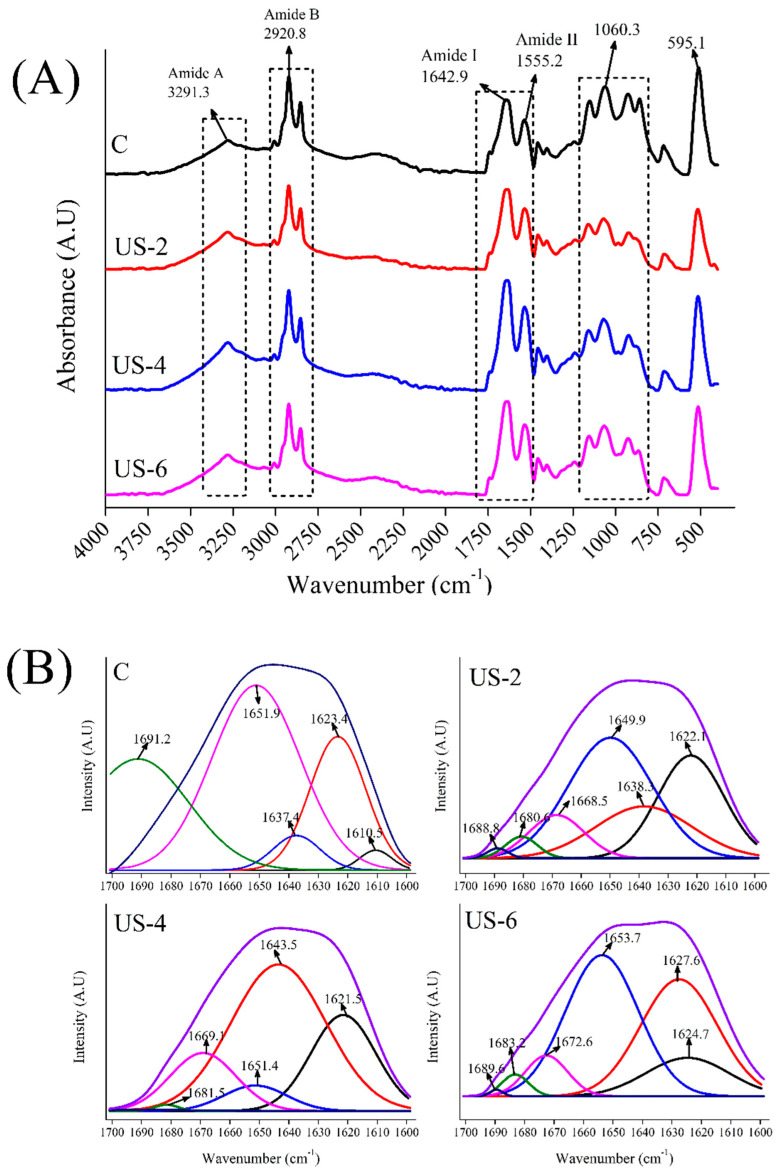
FTIR spectra (**A**) and related peak areas of untreated and high-intensity-ultrasound-treated sesame protein isolates (**B**). (C, untreated (control); US−2, US−4, and US−6, high-intensity ultrasound treatment for 2, 4, and 6 min, respectively).

In order to elucidate the changes in the structural properties of sesame protein isolates via ultrasound treatment with varying time, FTIR was used to determine the back-bone structure of control and ultrasonicated protein samples, and the FTIR spectrum is displayed in Figure 3a. As seen in Figure 3A, the FTIR spectra of the samples were very similar to each other. The ultrasound process led to a partial increase in the intensity of the amide A band. This is related to the increase in H bond formation with the sonication process [37]. Although there was no significant change in the wavelengths of the amide I and amide II bands, an increase in the intensity of the peaks was observed due to the ultrasound treatment. This indicates that changes occur in the secondary structural properties of proteins with the ultrasonication process. While the intensity of the band observed at the 1060.3 cm^−1^ wavelength in the control sample was high, the intensity of this band decreased with the ultrasound treatment. In a study carried out by Nazari, Mohammadifar, Shojaee-Aliabadi, Feizollahi, and Mirmoghtadaie [37], various changes were observed in the FTIR spectrum band intensities of millet proteins, which is associated with a reduction of intermolecular hydrogen bonds, a change in zeta potential, and a dispersion of particles.

In order to determine the effect of ultrasound treatment on the secondary structural properties of sesame protein isolates, the amide I band was further analyzed by Fourier self-deconvolution, and related peak areas are given in Figure 3b; as well, calculated peak areas are summarized in Table 1. Due to its remarkable sensitivity to even the smallest changes in molecular geometry and hydrogen bonding patterns, the amide I band is particularly helpful for examining the secondary structure of proteins. The secondary structure of proteins can be evaluated by the α-helix, β-sheet, β-turn, and random coil peaks ranging between the wavelengths of 1650–1658 cm^−1^, 1610–1640 cm^−1^, 1660–1700 cm^−1^, and 1640–1650, respectively [41]. In general, -CO and NH- groups form intramolecular hydrogen bonds to form α-helix structures, while polypeptide chains form inter-chain hydrogen bonds to stabilize β-sheets structures. The β-turn structures are formed by weakly hydrogen-bonded structures, whereas unfolded conformation corresponds to random coils and are related to protein flexibility [42]. As seen in Figure 3b, the wavenumbers and intensity of peaks related to secondary structures of proteins were significantly changed, probably due to the destroying of hydrogen-bonded structures by ultrasonication. The application of ultrasound at various times caused the unfolding of proteins as a response to the mechanical forces and cavitation effects of ultrasound processing [42].

The untreated sample displayed the highest α-helix content, and the lowest β-sheet, β-turn, and random coil (Table 1). The application of ultrasound decreased the α-helix content while it increased the β-sheet, β-turn, and random coil contents, showing that the increasing ultrasound time caused the transformation of α-helix to other components. The increasing β-sheet and decreasing α-helix content with ultrasound were also reported by Wang et al. [43] for egg yolk proteins and Li, Wang, Zheng, and Guo [42] for fish myofibrillar proteins, respectively. In a previous study [44], significant correlations were observed between the secondary structure and functional properties of soy protein isolates, revealing that increasing β-sheet content enhanced the emulsifying ability and stability. In this study, the increasing emulsifying properties with increasing ultrasound time could be attributed to increasing β-sheet content of sesame protein isolates. In addition, the decreasing α-helix content with increasing ultrasound treatment could be related to the increasing surface hydrophobicity, as reported earlier for bovine serum albumin [45].

### 3.4. Optical and Scanning Electron Microscopy (SEM)

**Figure 4:**
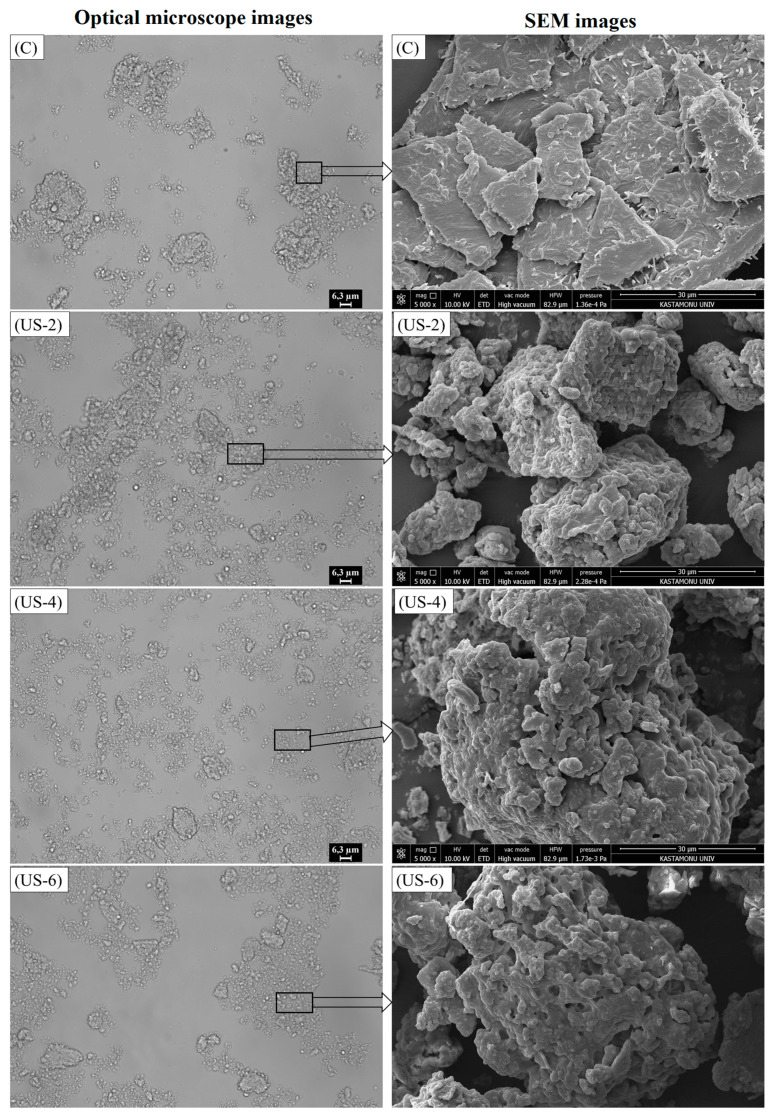
Optical microscope and SEM images of untreated and high-intensity-ultrasound-treated sesame protein isolates. (C, untreated (control); US−2, US−4, and US−6, high-intensity ultrasound treatment for 2, 4, and 6 min, respectively).

Figure 4 shows the optical micrographics of untreated and ultrasonicated sesame protein suspensions. According to microscope images, a clear difference was observed between control and ultrasonicated samples. The control sample mainly consisted of big, deformable particles with heterogeneous shapes, probably from flocculated particles, whereas a dispersed structure with smaller particles was obtained after the sonication process for 2 and 4 min, consistent with the particle size results. Moreover, a completely homogeneous microstructure was obtained after 4 min of ultrasonication. The increased sonication time from 4 to 6 min tended to cause small particles to be aggregated. However, when compared with the control group, it can be said that the proteins form a dispersal close to homogeneous distribution. Gordon and Pilosof [46] also showed the effect of high-intensity ultrasound on the physical properties of whey protein particles; they stated that the surface of the particles becomes smooth, and many small particles are formed as a result of the breakup of the large ones, when compared with an untreated sample.

The SEM microstructures of the control and ultrasonicated proteins with different times are shown in Figure 4. Compared to the unmodified sesame protein isolate, it was determined that the surface character of the ultrasonicated protein isolates completely changed, and the particles showed more rough and irregular surfaces. The sonication time impacted the roughness and geometry of sesame protein isolates. With the increase of sonication time, more irregular and larger particle sizes were formed. In addition, sonication led to the formation of pores in the protein isolates. Similar observations were obtained in previous studies for quinoa seed [47] and black bean protein isolates [48] via ultrasound treatment. These microstructural changes can be explained by the cavitation force applied by the sonication probe, and the micro jets and turbulence that occur during the high-intensity ultrasound process [47]. Zou et al. [49] stated that changes might occur in the microstructure of proteins due to the destruction of hydrogen bonds and van der Waals forces between proteins by local micro jets and oscillation waves formed by ultrasonic cavitation. The formation of cavitation or bubbles during sonication leads to the formation of more sulfhydryl and hydrophobic areas on the surface of the proteins [50], which are discussed below.

It is also noteworthy that the sonication process causes a decrease in particle size of protein isolates, which is consistent with the results of particle size. However, an increase in particle size was observed depending on the increase in sonication process time. These findings were in agreement with studies conducted by Ding, Tian, Wang, Deng, Mao, and Sang [33] and Karra et al. [51]. Zou, Shi, Chen, Xu, Jiang, Xu, and Wang [50] also reported that the protein molecular structure breakdown, particle size reduction, protein aggregate dispersion, and creation of looser layer structures after lyophilization are all potential effects of ultrasound treatment. With the reduction of particle size, the contact area of protein and water increases; hence, the solubility and hydrophobicity of proteins improve [33].

On the other hand, ultrasonic power higher than 500 W or long sonication time cause an increase in particle size [33,51]. It was observed that increasing the sonication time from 2 to 4 min led to a relative increase in particle size, and this increment was significant (*p* < 0.05) when the sonication time was increased to 6 min. Resendiz-Vazquez et al. [52] reported that a significant increase in the particle size of chicken myofibrillar protein isolate was observed as a result of the long-term application of sonication. The increase in particle size is thought to be due to the increase in charges during sonication and the emergence of free −SH and hydrophobic groups that can interact with each other on the surface of protein molecules and form larger aggregates during freeze drying [51].

### 3.5. Protein Solubility

Protein solubility, which plays a vital role in the functional properties (such as emulsification, thickening, and gelation) of proteins, is directly related to the denaturation and/or aggregation properties of proteins. The effect of sonication treatment on the solubility of sesame proteins is displayed in Table 2. Compared to the untreated sample, the solubility value was increased after sonication, and a maximum solubility of 80.73% was obtained after 6 min of sonication (*p* < 0.05). However, there was no remarkable change in the value of solubility after ultrasound treatment for 2 min (*p* > 0.05). Protein molecules have hydrophobic and hydrophilic groups that can interact with the water and oil phase, respectively. It is thought that an increase in solubility with ultrasound treatment is due to the effect of hydrophobic and polar groups in the proteins, and the change in particle size may increase the interaction between water molecules and proteins. Zhao, Liu, Liu, Liu, Zhang, and Hu [15] stated that conformational changes in the protein structure occurred during ultrasonication, resulting in smaller aggregates of proteins and exposing the hydrophilic regions to water; therefore, interaction areas between the water molecules and proteins increase, and insoluble precipitates turn into soluble protein molecules. The solubility results in this research are consistent with those of a study by Zhu, Zhu, Yi, Liu, Cao, Lu, Decker, and McClements [29], who stated that although no significant difference is observed in the solubility of walnut proteins between the untreated and treated samples sonicated at low power levels at different sonication times, the solubility values increase significantly when higher power levels and sonication times are used. In another study, in which soy proteins were subjected to ultrasonication at 80 W/cm^2^ for 0, 10, and 25 min, it was determined that the solubility of the soybean proteins increased with the increase in the processing time, and researchers revealed that high-intensity ultrasound can be effective at improving the functional properties of soybean proteins [53].

### 3.6. Surface Hydrophobicity

The amount of hydrophobic groups that are in contact with the polar aqueous medium on the surface of the protein molecule is determined by surface hydrophobicity, which is also directly related to the protein’s functional characteristics [24]. Due to the macromolecular structure of the protein, surface hydrophobicity rather than the total hydrophobicity value is more effective for protein functionality [54]. Thus, the surface hydrophobicity of untreated and ultrasonicated sesame proteins was determined and the results presented in Table 2. A significant change occurred in the surface hydrophobicity of the sesame protein after ultrasound treatment (*p* < 0.05). Sonication caused an increase in the hydrophobicity of proteins as compared to the control sample. Stathopulos et al. [55] reported that the surface hydrophobicity of the bovine serum protein increased with sonication treatment. In another study, Ren et al. [56] stated that the surface hydrophobicity value of the soy protein isolate after ultrasound treatment increased due to the decomposition of large aggregates in the protein isolate. This increment in surface hydrophobicity was attributed to the promotion of macromolecular aggregates due to the high intensity of shear forces, micro jets, shock waves, and turbulence induced by ultrasound waves, thereby exposing partially buried interior hydrophobic amino acid residues. Unfolding of the protein can facilitate its increased digestibility and interaction with enzymes [32]. This was increased when sonication time was increased up to 4 min; however, a decrease was detected as a result of increasing the treatment time to 6 min. The same outcomes were also determined by Malik, Sharma, and Saini [31], who reported that the reason for the decrease in surface hydrophobicity in long-term sonication is the loss of hydrophobic regions of proteins with protein aggregation. Yanjun, Jianhang, Shuwen, Hongjuan, Jing, Lu, Uluko, Yanling, Wenming, Wupeng, and Jiaping [35] reported that proteins that are partially denatured after prolonged sonication might undergo intense binding and reduction in surface hydrophobicity.

### 3.7. Free and Total Surface Sulfhydryl (−SH) Groups

The free and total −SH groups of sesame protein isolates are given in Table 2. As a result of sonication treatment, an increase in free and total −SH groups of sesame protein isolates was detected as compared with the control sample. This increase was not significant (*p* > 0.05) for the sample that was sonicated for 2 min (*p* > 0.05), but it was found to be statistically significant (*p* < 0.05) in the samples sonicated for a longer time. In the control sample, the free and total −SH group was 4.56 and 12.74 µmol/g protein, while they increased up to 7.04 and 14.94 µmol/g protein with ultrasound treatment, respectively. Similar results have been obtained in protein modification studies by ultrasound [15,50], and the possible explanations were attributed to the breaking of some S-S bonds, and structural instability as a result of ultrasound-induced cavitation, which allows for the formation of new hydrogen bonds [4]. In addition, the reduction in particle size during ultrasound due to the high pressure, turbulent flow, and shear force of the cavitation phenomenon may also play a role in the exposure of embedded sulfhydryl groups of protein [57]. In contradiction to these studies, a reduction in the sulfhydryl content of proteins was observed as an effect of ultrasound treatment. Gulseren, Guzey, Bruce, and Weiss [45] reported that ultrasound at 20 W/cm^2^ for 15, 30, and 45 min causes a reduction in the free −SH group of bovine serum albumin, while Chandrapala et al. [58] reported that high-intensity ultrasound (using an ultrasonic probe at 50% amplitude of 20 kHz at 450 W for 1, 5, 10, 20, 30, and 60 min) had no effect on the free −SH group of whey protein concentrate. In other study conducted by Amiri et al. [59] and Zhang, Regenstein, Zhou, and Yang [38], the total −SH group was found to decrease after ultrasound treatment, probably due to the formation of disulfide bonds, and a reverse trend was observed for free −SH groups that could be related to the disruption of protein structure and reduction in particle size. Kahraman et al. [60] stated that the reduction in free −SH groups could be associated with the oxidation of disulfide bonds with hydrogen peroxide, which forms by the generation of free radicals during sonication.

### 3.8. Emulsifying Properties

The emulsion activity index (EAI) and stability index (ESI), which positively correlate with protein solubility, hydrophobicity, and conformational flexibility, were used to determine the emulsifying properties of sesame proteins. The EAI reflects the ability of proteins to quickly adhere to the water–oil interface during emulsion formation. The ESI is the ability of the proteins to maintain the stability of emulsions to which they are added for a certain period. The effect of ultrasound on the emulsifying properties of sesame protein dispersions is shown in Table 2. The EAI of the untreated sesame protein isolate was determined as 31.42 m^2^/g, and it was significantly (*p* < 0.05) enhanced up to 38.85 m^2^/g with the application of ultrasound. Similarly, the ESI in the control sample was determined as 51.09 min, reaching 77.01 min with ultrasound treatment at 6 min. The highest EAI and ESI values were obtained in sesame proteins treated by ultrasound for 6 min. The enhancement of emulsifying properties of the sesame protein is explained by the fact that ultrasound led to changes in the surface chemistry of the proteins and the formation of smaller soluble proteins absorbedin the oil–water interface [4,15]. Our results are consistent with previous studies on potato [15] and almond [61] protein isolates, showing an improvement in emulsifying properties as a result of ultrasound treatment.

### 3.9. Foaming Properties

Proteins’ functional qualities of foaming, such as capacity (FC) and stability (FS), are critical for the puffing of food and foamed beverages because they affect the mellowness, softness, smoothness, and brightness of the meal [33]. In general, ultrasound treatment led to enhancement of the foaming properties (*p* < 0.05, Table 2). The FC and FS of untreated sesame protein was 83.95% and 20.71%, respectively, and the maximum FC (160.19%) was observed in the sample sonicated for 4 min, while the maximum FS was determined as 86.13% in the isolate ultrasonicated for 6 min. Similar findings were observed in pumpkin seed [14] and scallop [33] protein isolates. It has been stated that the increase in foam properties with the ultrasonication process may be due to the partial denaturation of proteins, the formation of a high water–air diffusion interface, and an increase in the adhesion and flexibility of the foam [62]. In another study, it was determined that the formation of small and uniform gas bubbles with the ultrasound process improves the foam properties. It has been stated that this situation is caused by the partial dissolution of the protein structure, the decrease in the particle size of the protein particles, the formation of hydrophobic amino acid residues, and the adsorbing of viscoelastic films on the water–air interface [30].

### 3.10. Water- and Oil-Holding Capacity

The water- and oil-holding capacities of the control and ultrasonicated sesame protein isolates are given in Table 2. The water- and oil-holding capacities of the untreated sesame protein isolate were determined as 1.83 g water/g protein and 1.39 g oil/g protein, respectively. As seen in Table 2, the water-holding capacity of sesame protein increased as the ultrasonication treatment increased to 4 min. Then it decreased when the sonication time reached 6 min. It is predicted that the increase could be attributed to the improvement of the water solubility of the proteins and the increased surface area of the proteins with the decrease in particle sizes, resulting in a combination of hydrophilic groups of proteins and water molecules [33]. Similar results were reported for soybean protein isolate by Hu, Li-Chan, Wan, Tian, and Pan [57], who found that samples treated with high-intensity ultrasonic treatments for up to 40 min led to an increase in water-holding capacity. The decrease in water-holding capacity after sonication for 6 min could be explained by denaturation of the molecular structure of sesame protein isolate, with a similar result obtained by Mir, Riar, and Singh [47] for quinoa protein isolates sonicated for a longer duration time.

The positive effect of ultrasound on the oil-holding capacity of the samples was observed, and it increased to 1.61 g oil/g protein at 6 min. It is thought that the increase in oil-holding capacity may be due to the partial dissolution of protein structures and exposure of hydrophobic groups on the surface, promoting network formation in a structure that will entrap oil droplets [63].

### 3.11. Rheological Properties

#### 3.11.1. Steady Shear Properties

**Figure 5:**
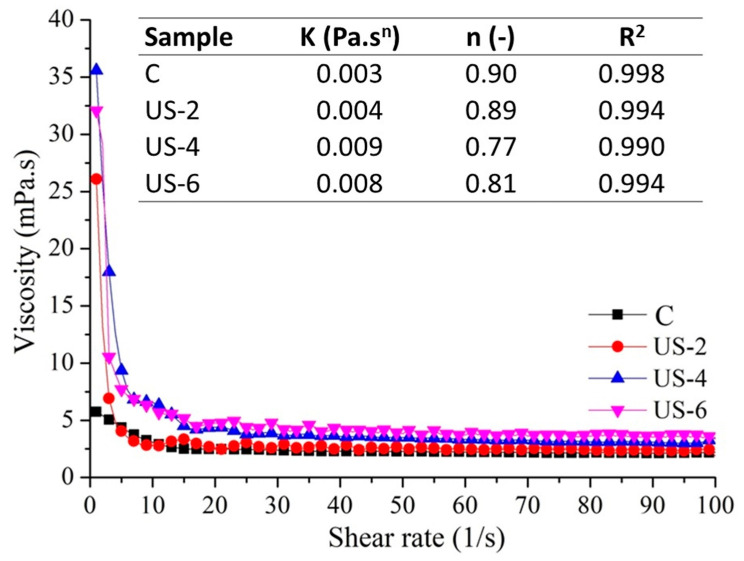
Steady-state shear properties of untreated and high-intensity-ultrasound-treated sesame protein isolates. (C, untreated (control); US−2, US−4, and US−6, high-intensity ultrasound treatment for 2, 4, and 6 min, respectively).

The flow behavior of sesame protein solutions before and after ultrasound treatment is shown in Figure 5. Sesame protein suspensions exhibited shear-thinning or pseudo-plastic behavior due to decreasing viscosity with increasing shear rates, which was reported earlier by Di, Li, Chang, Gu, Duan, Liu, Liu, and Wang [8] for sesame protein and by Saricaoglu [64] for lentil protein. The lowest shear force curve was determined in the control samples, while the application of ultrasound led to increasing shear force values at increasing shear rates. As can be seen from the viscosity–shear rate graph, the lowest viscosity values were determined in the control group, while increasing ultrasound treatment time caused a significant (*p* < 0.05) increase in the viscosity of the sesame proteins. A similar trend was observed for date palm pollen protein concentrate by Sebii et al. [65], who reported that the sonicated protein concentrate is characterized by the highest viscosity values of all the tested solutions. This was due to the increase of water-soluble protein, decreased protein–protein interactions, and increased protein–water interactions induced by ultrasound, which is described in water-holding capacities. However, Amiri, Sharifian, and Soltanizadeh [59] reported that the viscosity values of myofibrillar proteins decreased with the application of ultrasound treatment, which could be related to a breaking of the bonds between the myofibrillar protein strands and a decrease in particle size due to physical forces formed during cavitation [21]. In the stress vs. rate mode, the Ostwald–de Waele model was successfully used to described the flow curves of protein solutions, as indicated by R^2^ values higher than 0.99, and the changes in the flow behavior index (*n*) and consistency coefficient (K) of untreated and ultrasonicated sesame protein dispersions are shown in Figure 5. The *n* values of all samples were lower than 1, which is corrected to shear thinning flow behavior. The lowest K value and highest *n* value were obtained for the control sample, which meant the lowest viscosity. As the sonication time increased from 2 to 4 min, the *n* value was reduced but the K value increased, which was consistent with the outcomes formerly reported by Sebii et al. [65] for date palm pollen protein and Jambrak et al. [66] for soy protein concentrates.

#### 3.11.2. Dynamic Shear Tests

**Figure 6:**
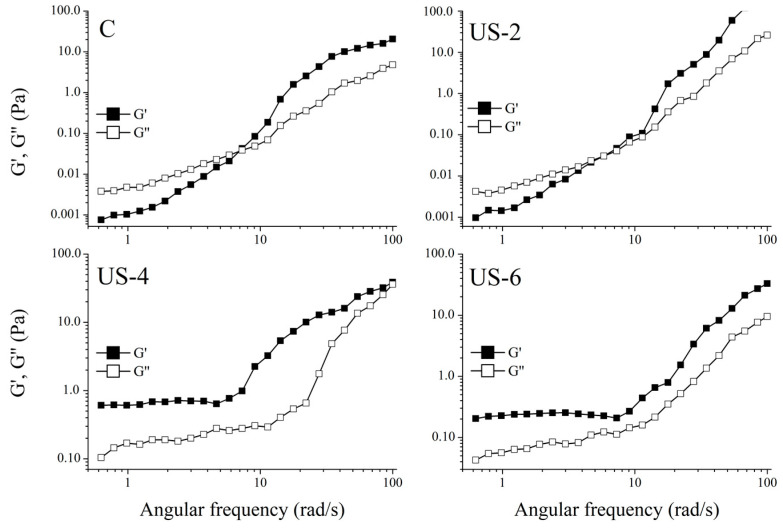
Dynamic shear properties of untreated and high-intensity-ultrasound-treated sesame protein isolates. (C, untreated (control); US−2, US−4, and US−6, high-intensity ultrasound treatment for 2, 4, and 6 min, respectively).

Figure 6 displays the linear frequency sweep results showing the change in the *G*′ and *G*″ moduli for the sesame protein solutions treated with ultrasound for up to 6 min over a range of 0.01 to 10 rad/s. It was found that both *G*′ and *G*″ increased when increasing the frequency for all samples, which was indicated by a high-frequency dependency. Similar trends were reported earlier for various types of protein solutions [24,65]. As seen in the figure, the viscous character was dominant in the samples that were untreated and ultrasonicated for 2 min. However, the viscoelastic structure became dominant with the increase in frequency. In addition, the viscoelastic (gel-like) structure became dominant at a lower frequency when the sample was treated for 2 min compared to the control sample. On the other hand, *G*′ values of samples ultrasonicated for 4 and 6 min were much higher than *G*″ over the frequency range studied, indicating a viscoelastic structure typically observed in gels. This situation also coincides with the apparent viscosity results. Although the 4% protein concentration for sesame proteins is determined to be low in terms of obtaining a viscoelastic structure, a viscoelastic structure could be obtained even at low protein concentrations by sonication. This is thought to be due to the strong cavitation induced by the ultrasound and the changes in the surface properties of proteins. In addition, as can be seen from the microstructure images, the increased sonication treatment time caused a better dispersion of the proteins in the suspended state, reduced particle sizes, and thus contributed to the development of the viscoelastic structure.

The storage and loss moduli of the samples were well modeled with the power law function (*R*^2^ > 0.970) to determine the dependency of the modulus on the angular frequency. As seen from the Table 3, all the $K^{\prime }$ values were higher than $K^{\prime \prime }$, indicating viscoelastic structure, even if protein suspensions for the control and US−2 displayed a viscous character at lower frequencies. The $G^{\prime \prime }$ showed a higher dependency to angular frequency because of higher $n^{\prime \prime }$ values than $n^{\prime }$. The increasing ultrasound time caused a viscoelastic structure and, hence, decreased the frequency dependency of protein suspensions. It can also be inferred from these results that ultrasound treatment at low protein concentrations led to the producing of a viscoelastic gel-like structure.

#### 3.11.3. Temperature Sweep Tests

**Figure 7:**
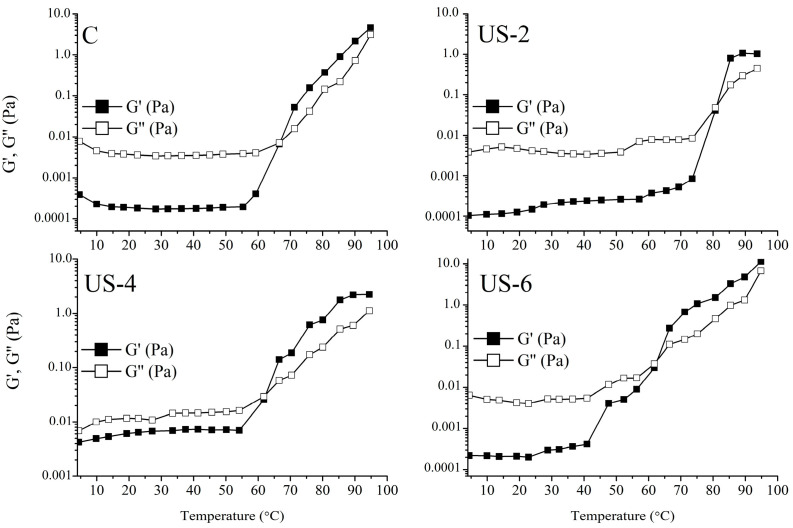
Temperature sweep results of untreated and high-intensity-ultrasound-treated sesame protein isolates. (C, untreated (control); US−2, US−4, and US−6, high-intensity ultrasound treatment for 2, 4, and 6 min, respectively).

Temperature dependency of untreated and ultrasonicated sesame protein solutions and the effect of ultrasonication on the gelation temperature of proteins were determined, and the results of the temperature sweep test are shown in Figure 7. It was observed that *G*″ values of all samples were higher than *G*′ at low temperatures, implying a liquid structure. However, the *G*′ value of each sample was significantly increased with increasing temperature above 60 °C and was higher than the G’’ value, indicating the dominance of the viscoelastic structure. For control samples, a gel formation was observed at about 70 °C, which was obtained from a *G*′ and *G*″ cross-over. A significant increase (80 °C) in gelation temperature was determined for the sample ultrasonicated for 2 min. On the contrary, ultrasound treatment for 4 and 6 min caused a decrease in the gelation temperature of proteins at around 60–65 °C. The determined difference in the gelation temperature was probably due to the changes in the structure and surface hydrophobicity of the proteins via the ultrasound process. Xiong et al. [67] investigated the gel formation state of ovalbumin proteins treated with high-intensity ultrasonication with temperature and found that the gelation shifted towards higher temperatures with ultrasonication. Researchers have stated that this difference is attributable to the reduction of sulfhydryl bonds into disulfide bonds, and, thus, the change in thermal and rheological properties. In another study, the effect of ultrasonication on soy protein gelling was examined, and it was found that the ultrasonicated samples had a faster heating and cooling tendency and higher gelling temperatures [24].

## 4. Conclusions

Recently, due to the increasing trend toward plant proteins, studies on plant proteins have increased, and the plant-derived proteins still have lower functional properties than proteins of animal origin. That is why modification studies aimed at improving the functional properties of plant proteins have gained importance. In this study, sesame seed cake, an oil industry by-product, was used for protein extraction, and the effects of ultrasound treatments on the functional, chemical, and rheological properties of sesame protein isolate were further investigated. Our findings revealed that an increased ultrasound time caused increased solubility and decreased particle size, as well as enhanced the functional properties when compared to the control sample. The secondary structure of proteins significantly changed, and the results were well related to the surface properties. Additionally, high-intensity ultrasound resulted in significantly (*p* < 0.05) increased surface hydrophobicity and free and total −SH groups. Moreover, the viscosity and viscoelastic structure of protein suspensions were improved even at low concentrations (4%). In general, it can be concluded from these results that ultrasound treatment for 4 and 6 min could be useful for obtaining a better sesame seed protein suspension for the utilization in the food industry.

## Figures and Tables

**Table 1:** Secondary structures of untreated and high-intensity-ultrasound-treated sesame protein isolates.

Sample	α-Helix (%)	β-Sheet (%)	β-Turn (%)	Random Coil (%)
C	45.58	20.45	17.65	16.32
US−2	35.53	26.55	19.11	18.81
US−4	21.15	33.82	20.91	24.12
US−6	16.80	34.53	21.72	26.95

C, untreated (control); US−2, US−4, and US−6, high-intensity ultrasound treatment for 2, 4, and 6 min, respectively.

**Table 2:** Physical and functional properties of untreated and high-intensity-ultrasound-treated sesame protein isolates.

Properties	C	US−2	US−4	US−6
Protein solubility (%)	54.72 ± 2.31 ^c^	56.94 ± 1.33 ^c^	66.29 ± 3.88 ^b^	80.73 ± 3.29 ^a^
Surface hydrophobicity (H_0_)	66.72 ± 2.18 ^c^	93.88 ± 3.42 ^ab^	100.61 ± 3.92 ^a^	89.48 ± 2.41 ^b^
Free −SH group (µmol/g)	4.56 ± 0.04 ^c^	4.76 ± 0.03 ^c^	5.22 ± 0.08 ^b^	7.04 ± 0.34 ^a^
Total −SH group (µmol/g)	12.75 ± 0.79 ^c^	12.99 ± 0.28 ^c^	13.75 ± 0.19 ^b^	14.94 ± 0.39 ^a^
EAI (m^2^/g)	31.42 ± 0.51 ^c^	33.68 ± 0.61 ^b^	37.49 ± 0.73 ^a^	38.85 ± 0.8 ^a^
ESI (min)	51.09 ± 2.18 ^c^	60.71 ± 1.62 ^b^	65.94 ± 2.19 ^b^	77.01 ± 3.62 ^a^
FC (%)	83.95 ± 5.45 ^d^	113.34 ± 5.17 ^c^	160.19 ± 3.57 ^a^	151.34 ± 4.21 ^b^
FS (%)	20.7 ± 2.72 ^c^	30.46 ± 2.48 ^b^	79.17 ± 4.72 ^a^	86.13 ± 3.2 ^a^
Water-holding capacity (g water/g)	1.83 ± 0.09 ^c^	1.9 ± 0.18 ^bc^	2.4 ± 0.16 ^a^	2.02 ± 0.08 ^b^
Oil-holding capacity (g oil/g)	1.39 ± 0.13 ^b^	1.46 ± 0.13 ^ab^	1.58 ± 0.12 ^a^	1.61 ± 0.12 ^a^

^a–d^ Means within the same line with different letters are different (*p* < 0.05). C, untreated (control); US−2, US−4, amd US−6, high-intensity ultrasound treatment for 2, 4, and 6 min, respectively. EAI: emulsion activity index; ESI: emulsion stability index; FC: foaming capacity; FS: foaming stability.

**Table 3:** Dynamic shear parameters of power law functions describing *G*′ and *G*′′ values of untreated and high-intensity-ultrasound-treated sesame protein isolates.

	$G^{\prime }=K^{\prime }{\left(\omega \right)}^{n^{\prime }}$			$G^{\prime \prime }=K^{\prime \prime }{\left(\omega \right)}^{n^{\prime \prime }}$		
	$K^{\prime }$ (×10^−2^ Pas^n^)	$n^{\prime }$	*R* ^2^	$K^{\prime \prime }$ (×10^−2^ Pas^n^)	$n^{\prime \prime }$	*R* ^2^
C	9.61	1.17	0.972	0.44	1.52	0.994
US-2	1.96	2.01	0.970	0.08	2.27	0.991
US-4	40.65	0.99	0.990	0.61	1.89	0.994
US-6	1.90	1.63	0.991	0.49	1.65	0.991

C: Control; ***K***′ and ***K***″: constants; ***n***′ and ***n***″: frequency exponents; ***R*^2^**: determination coefficient. C, untreated (control); US−2, US−4, and US−6, high-intensity ultrasound treatment for 2, 4, and 6 min, respectively.

## Data Availability

All data generated and/or analyzed during this study are available from the corresponding author on reasonable request.
